# Development and validation of a scale to measure hypertensive Chinese individual’s behavior toward salt reduction consumption

**DOI:** 10.1186/s12889-024-18925-9

**Published:** 2024-05-31

**Authors:** Le Han, Ying Liu, Xiao Liu, Peng Xian

**Affiliations:** Hospital Infection Management & Disease Control and Prevention Department, Beijing Tongren Hospital, No.1 Dongjiaominxiang, Beijing, China

**Keywords:** Salt reduction, Hypertension, Health Belief Model (HBM), Behavioral scale, Confirmatory Factor Analysis (CFA)

## Abstract

**Objective:**

This study aimed to develop and validate the Salt Reduction Behavior Scale (SRBS) to measure the behavior of hypertensive Chinese individuals in adhering to salt reduction practices.

**Methods:**

The SRBS was constructed based on the Health Belief Model, consisting of five constructs: knowledge, perceived severity, perceived benefits, perceived barriers, and cues to action. Data were collected from 2,082 hypertensive patients in Beijing, China, who completed a questionnaire administered through an online platform. Kaiser-Meyer-Olkin (KMO) test was used to assess the adequacy of the sample and the Bartlett’s test of sphericity to examine the factorability of the dataset. Confirmatory Factor Analysis (CFA) was used to assess the structural validity and reliability of the SRBS.

**Results:**

The KMO analysis yielded a notably elevated value of 0.95, indicating that the data was highly suitable for Exploratory Factor Analysis (EFA). Bartlett’s test of sphericity yielded a statistically significant test statistic (*P* < 0.001). The 32-item SRB questionnaire demonstrated strong internal consistency with a Cronbach’s alpha coefficient of 0.923. A second-order Confirmatory Factor Analysis (CFA) revealed that, after removing the unrelated construct of barriers, SRB could be represented by four sub-constructs: knowledge, severity, benefits, and action. The final version of the SRBS consists of 21 items. These items displayed high factor loadings, indicating a strong relationship between the items and their respective sub-constructs. The discriminant validity analysis revealed that the SRBS sub-constructs were distinct from each other. The SRBS scores were positively correlated with self-reported salt reduction practices. This demonstrates that individuals with higher SRBS scores were more likely to engage in actual salt reduction behaviors, indicating concurrent validity.

**Conclusion:**

The results illustrate that the Salt Reduction Behavior Scale is a robust and comprehensive instrument for assessing salt reduction behavior among hypertensive Chinese individuals. The scale’s specific sub-constructs provide a detailed understanding of their knowledge, attitudes, and practices related to salt consumption. Healthcare professionals and policymakers can utilize this tool to tailor interventions and educational programs to encourage healthier dietary habits, thereby reducing the risk of cardiovascular diseases in China.

## Introduction

The global prevalence and ominous nature of non-communicable diseases (NCDs) represent a formidable challenge within the realm of public health. These illness stand as foremost contributors to worldwide mortality rates and inflict a substantial socioeconomic toll, effectively entrapping individuals in the cycle of poverty [1]. Elevated salt consumption is intricately linked with the elevation of blood pressure, a pivotal risk factor in the development of cardiovascular diseases, notably encompassing heart disease and strokes [2, 3]. Numerous interventional and observational studies have diligently explored the intricate relationship between dietary sodium intake and blood pressure levels over recent decades. Consequently, an accumulating body of empirical evidence substantiates a direct correlation between sodium intake and the augmentation of blood pressure [4].In accordance with the 2012 guidelines set forth by the World Health Organization (WHO), it is recommended that adults aged 16 years and above limit their daily sodium intake to less than 2000 mg, which is equivalent to consuming less than 5 g of salt per day [5]. Modest yet sustained reductions in dietary salt intake hold the potential for substantial public health dividends. Even a relatively modest reduction in blood pressure resulting from diminished sodium intake across the entire population, including normotensive individuals, may culminate in a noteworthy reduction in the incidence of cardiovascular diseases [6]. Numerous nations have embarked upon comprehensive salt-reduction strategies, encompassing multifaceted approaches such as public health education, reformulation of processed food items, and the implementation of front-of-pack nutritional labeling initiatives [6, 7].

Population-wide reduction of salt intake stands out as a highly cost-effective intervention for the prevention and management of elevated blood pressure, thereby mitigating the risks of associated health conditions, notably cardiovascular disease [8].In China, the prevailing levels of salt consumption are a cause for concern, averaging between 12 and 14 g per person daily, surpassing the World Health Organization’s recommended daily maximum intake of 5 g [9]. The burden of high blood pressure is significant, affecting over 244 million Chinese adults, alongside another 435 million grappling with pre-hypertension. Alarmingly, elevated blood pressure contributes to an excess of 2 million cardiovascular disease-related deaths within China [10].

Reformulation strategies are expected to yield heightened effectiveness, particularly in nations where a substantial portion of dietary salt is derived from processed and fast foods. Notably, research has demonstrated that the primary source of dietary salt in the Chinese population is salt added during home-cooking, a contrast to the situation in Western countries where processed and fast foods are the major sodium contributors [11]. Consequently, concerted salt reduction efforts in China should prioritize self-awareness education to motivate individuals to curtail salt usage in their culinary practices [12]. However, altering behavior in this context poses a formidable challenge; short-term dietary interventions aimed at salt reduction have historically failed to yield sustained, long-term results. Additionally, adherence to a salt-restricted diet has proven crucial, as poor compliance was significantly associated with uncontrolled blood pressure increases.

Health education is widely recognized as an effective intervention strategy for reducing salt intake. However, there exists an evident gap in understanding the underlying reasons for the reluctance among individuals in China to adopt salt-restriction diets [13]. A comprehensive exploration of the psychosocial determinants of this behavior is imperative. Such insights can significantly contribute to informing health education professionals in the development of nuanced policies and strategic interventions that facilitate adherence to salt restriction behaviors. In this research endeavor, our primary objective was the formulation and validation of a Salt-Restriction Behavior Scale (SRBS), tailored for the assessment of salt-restriction dietary practices among hypertensive patients in China. The development of scales of this nature serves the purpose of quantifying latent constructs related to behaviors and attitudes that inherently elude direct measurement. The conceptual framework for the SRBS draws inspiration from the Health Belief Model (HBM), a well-established theoretical construct within the domain of health behavior analysis [14].

The Health Belief Model (HBM) offers a comprehensive theoretical lens through which to examine individual perceptions concerning the gravity of health threats, encompassing dimensions of susceptibility and severity. Additionally, it considers the perceived advantages of engaging in preventive actions, alongside the various determinants influencing the decision-making process, including perceived barriers, cues to action, and self-efficacy. Originating in the 1950s, this model was developed by a group of social psychologists within the U.S. Public Health Service, with the primary objective of elucidating the rationale behind the limited participation of individuals in programs geared towards disease prevention and detection [14].

Hypertension, often referred to as the “silent killer” due to its typically asymptomatic presentation, poses a substantial health challenge. According to the tenets of the HBM, individuals devoid of symptoms may be disinclined to adhere to prescribed treatment regimens unless they recognize their susceptibility to hypertension. Moreover, an appreciation of the condition’s severity, including its potential to precipitate adverse cardiovascular events such as heart attacks and strokes, is deemed crucial. Understanding the perceived benefits of compliance with prescribed medications and health programs, particularly when devoid of negative side effects and excessive complexities, plays an instrumental role in fostering adherence, as outlined by the HBM.

The application of the Health Belief Model proves highly pertinent in contexts characterized by noncompliance with recommended health behaviors. In this research initiative, HBM has provided the theoretical underpinning for the development of the Salt-Restriction Behavior Scale. Our aim has been to elucidate the psychosocial determinants influencing salt-restriction behaviors among hypertensive individuals in China. This endeavor holds substantial promise in informing the design and implementation of pragmatic and efficacious dietary intervention programs tailored to the needs of hypertensive patients. Furthermore, the SRBS serves as a valuable tool for the assessment of the effectiveness.

## Materials and methods

### Target population

The study focused on hypertensive patients selected from ten community healthcare service centers located within an urban district in Beijing. In accordance with rigorous inclusion criteria, eligible participants were required to meet the following conditions:

(a) Attainment of an age threshold surpassing 18 years. (b) Possession of Chinese citizenship. (c) A formal diagnosis of hypertension as determined by a tertiary-level hospital. (d) Proficiency in verbal, written, and reading comprehension of the Chinese language.

The research implemented well-defined exclusion criteria to enhance the precision of participant selection:

(a) Individuals demonstrating cognitive impairment were excluded from the study. (b) Participants experiencing communication difficulties stemming from functional impairments, such as blindness or deafness, were likewise ineligible for inclusion.

### Ethics statement

Prior to the commencement of the survey, explicit written informed consent was diligently secured from each participant, in strict adherence to the ethical principles set forth in the Helsinki Declaration. It is imperative to note that the questionnaire employed in this study was designed to ensure participant anonymity, refraining from the collection of directly identifying information, such as names, social insurance numbers, or personal health numbers. Furthermore, this study has undergone a thorough ethical review and has been granted approval by the Ethics Board of Beijing Tongren Hospital, affirming its compliance with ethical standards and guidelines.

### Sample size

This cross-sectional survey encompassed 21 community medical care service centers within an urban locale in Beijing. The sample size determination employed the formula:

$$n=\frac{{u}_{a}^{2}\pi \left(1-\pi \right)}{{\delta }^{2}}$$

where:

π represents the proportion of hypertensive individuals in this district, estimated at 20% of the urban hypertensive population in Beijing (i.e., 20%),

δ denotes the margin of error (i.e., 0.05),

and u_0.05 corresponds to the critical value at the 0.05 significance level (i.e., 1.96).

The initial sample size was computed as *n* = 246. However, considering an anticipated non-response rate of 20%, the final sample size was adjusted to *n* = 308.

In the context of constructing a path model, adherence to Klein’s recommendation of a minimum of 10 cases for each parameter estimated was observed. Given the total number of items encompassed in the self-designed questionnaire, a sample size of 340 was deemed adequate for each model under examination.

### Salt-restriction behavior scale questionnaire (SRBS)

The survey instrument, denoted as the Salt-Restriction Behavior Scale (SRBS), was methodically constructed with a theoretical underpinning grounded in the Health Belief Model (HBM). The SRBS functioned as a comprehensive tool to assess participants’ adherence to salt-restriction behaviors. Informed by previous scholarship on the intricate dynamics linking knowledge, attitudes, and dietary practices concerning salt consumption, the questionnaire was meticulously tailored to align with the sociocultural context of the Chinese population in Beijing.

The questionnaire was thoughtfully structured to encapsulate five distinct domains, namely:

Knowledge Pertaining to Hypertension and Salt Reduction

Perceived Severity of Hypertension

Perceived Benefits Derived from Salt Reduction

Perceived Barriers to Adhering to a Salt-Restriction Diet

Cues to Action: Factors likely to prompt individuals to engage in salt-reduction practices.

During the data collection phase, spanning from February to March 2022, the questionnaire was administered through an online platform. Respondents were prompted to assess the questionnaire items using a Likert scale, incorporating both 2-point and 5-point scales to comprehensively capture the nuanced aspects of participants’ responses.

### The survey instrument

The initial questionnaire featured a comprehensive set of 32 items thoughtfully distributed across five distinct constructs:

Construct 1: Knowledge Pertaining to Hypertension and Salt Intake (Incorporating 8 Items) This construct was designed to evaluate participants’ grasp of the correlation between hypertension and salt consumption.

Construct 2: Perceived Severity of Hypertension (Comprising 6 Items) Within this construct, participants’ perceptions regarding the gravity and potential ramifications of hypertension were assessed.

Construct 3: Perceived Benefits of Adhering to a Salt-Reduction Diet (Comprising 4 Items) This construct sought to elucidate participants’ perspectives on the advantages and favorable outcomes associated with the adoption of a salt-restricted dietary regimen.

Construct 4: Perceived Barriers to Practicing a Salt-Restriction Diet (Incorporating 5 Items) Questions within this construct were structured to elicit responses regarding the impediments and challenges individuals may encounter when endeavoring to adhere to a salt-restricted diet.

Construct 5: Cues to Action (Comprising 9 Items) This construct encompassed inquiries aimed at identifying factors and stimuli that could potentially motivate or prompt individuals to engage in behaviors aligned with salt-reduction practices.

### Hypotheses

Null Model: The null model, by definition, assumes that all correlations and covariances between variables are constrained to be zero.

Model 1: In this model, it is hypothesized that the five constructs comprising the study form five independent and uncorrelated first-order factors, with no interrelations among them.

Model 2: The second model posits that the five first-order factors exhibit significant correlations with each other, implying that there exist interdependencies and associations among these constructs.

Model 3: This model proposes that the first-order factors are linked to a singular second-order factor, denoted as “SRB.” This hypothesis suggests a hierarchical structural framework wherein the collective contributions of the first-order factors converge to influence the emergence and composition of this higher-level construct, SRB.

### Statistical analysis

Descriptive statistics summarizing the characteristics of the survey participants were methodically compiled and presented in tabular format, offering a comprehensive overview of the demographic and socio-economic attributes of the study’s respondents.

The validation of the model was conducted within the framework of Structural Equation Modeling (SEM), employing the Confirmatory Factor Analysis (CFA) method. This involved a comparison between two distinct models:

A first-order CFA model, often termed the measurement model scrutinized the relationships between observed variables and their corresponding latent constructs.

A second-order CFA model, referred to as the structural model, was used to explore the higher-level structural relationships among latent factors.

All statistical analyses were carried out using SPSS version 23.0 and Amos 23.0, ensuring a rigorous and methodologically sound assessment of the proposed models.

## Results

### Participant

We employed a convenience sampling approach to selectively recruit hypertensive patients from 21 primary healthcare facilities situated in an urban district of Beijing. A total of 2,450 hypertensive patients were initially enrolled for this study. However, within this pool, 278 individuals declined to participate, and an additional 90 did not complete the questionnaire. Consequently, a total of 2,082 participants satisfactorily completed the questionnaire.

Table 1 presents a comprehensive overview of the socio-demographic characteristics of the study participants. Of the enrolled individuals, 749 were male, constituting 36% of the overall surveyed population, while 1,333 were female, accounting for 64% of the surveyed population. The average age of all participants was calculated at 54.48 ± 14.78 years. The majority of participants were in a retired status and cohabiting with their partners, representing 54.2% and 54% of the total surveyed population, respectively.

Moreover, it is noteworthy that participants falling within the annual family income bracket of 3,001 to 6,000 yuan constituted 40.4% of the cohort.

**Table 1:** The characteristics of the participants

Characteristics		*n*%
Gender	Male	36(749)
	Female	64(1333)
Occupation	Retirement	52.4(1092)
	service industry	24.9(519)
	medicine and health industry	14.3(298)
	manufactory industry	4(84)
	Others	4.3(89)
Age	18–25	1.9(40)
	26–40	20.7(432)
	41–55	25.3(526)
	56–60	12.7(265)
	61–75	34.6(721)
	≥ 76	4.7(98)
Family revenue per month	≤ 3000	9.3(194)
	3001–6000	40.4(842)
	6001–9000	23.9(498)
	9001–12,000 14.6(304)	
	12,001–15,000	5.9(123)
	> 15,001	5.8(121)
Co-resident	Spouse	54(1124)
	Parents	17.3(361)
	Alone	10.3(215)
	Children	16.6(346)
	Others	1.7(35()
Hypertensive family history	Yes	30.5(636)
	No	69.5(1446)
Chronic disease	Yes	702(33.7)
	No	1380(66.3)

### Exploring factor analysis

In the course of our investigation into factor analysis, we administered both the Kaiser-Meyer-Olkin (KMO) test to assess the adequacy of the sample and the Bartlett’s test of sphericity to examine the factorability of the dataset. As presented in Table 2, the KMO analysis yielded a notably elevated value of 0.95, indicative of the data’s favorable suitability for Exploratory Factor Analysis (EFA) [15]. Additionally, the Bartlett’s test of sphericity yielded a statistically significant test statistic (*P* < 0.001), providing further support for the appropriateness of the dataset for EFA. It is noteworthy that this analysis was conducted utilizing SPSS software version 23.0.

**Table 2:** Kaiser-Meyer-Olkin (KMO) sample adequacy test

KMO and Barlett’s Test		
Kaiser-Meyer-Olkin Measure of Sampling Adequacy		0.951
Barlett’s Test of Sphericity	Approx.Chi-Square	60727.885
	Df	496
	Sig.	0.000

### Internal consistency

The 32-item SRB demonstrated a notable Cronbach’s alpha coefficient of 0.923, and it is worth emphasizing that the Cronbach’s alpha coefficients for all constituent constructs exceeded the conventional threshold of 0.7 see in Table 3, thereby affirming the presence of strong internal consistency, indicative of a high level of reliability within the constructs under examination [16, 17].

**Table 3:** Internal consistency

Constructs	Cronbach’s alpha	Items
Knowledge	0.829	8
Perceived of severity	0.941	6
Perceived of barriers	0.89	4
Perceived of benefits	0.966	5
Cue to action	0.963	9
SRB	0.924	32

### First-order confirmatory factor analysis

Confirmatory factor analysis (CFA) was employed to assess the degree to which the measured variables effectively represented underlying constructs. As delineated in Table 4, the results revealed that all fit indices fell within the acceptable range. Subsequently, following the exclusion of items with factor loadings below the threshold of 0.5, the ultimate model comprised five constructs encompassing 24 items. These constructs were subjected to a first-order confirmatory factor analysis, as presented in Table 4. The first construct, denoted as “Knowledge,” comprised five items, all of which exhibited factor loadings surpassing the recommended threshold of 0.5, as advocated by Awang [18]. Moreover, the t-values and associated p-values demonstrated statistical significance. In terms of internal consistency, all composite reliability (CR) values exceeded the criterion of 0.7, affirming a robust level of reliability, except for the “knowledge” construct. However, the average variance extracted (AVE) for the ‘Knowledge’ construct was below 0.5. Despite this, the CR value for ‘Knowledge’ was 0.763, indicating a sufficient level of reliability. Additionally, all other constructs had AVE values that exceeded 0.5, indicating acceptable convergent reliability. Furthermore, the square multiple correlation (SMC) values reported in Table 4 surpassed the acceptable threshold of 0.30 [19].

Discriminant validity was evaluated according to the methodology outlined by Fornell and Larcker [20], involving a comparison of the square root of the average variance extracted (AVE) along the diagonal with the correlation coefficients (off-diagonal) representing each construct in their respective rows and columns. The findings presented in Table 5 substantiate the established discriminant validity between the constructs within the measurement model.

**Table 4:** Results of CFA of 24-item SRBS (salt- restriction behavior scale) (Appendix A)

Constructs	Items					Convergent validity			
		Unstd.	S.E.	t-value	P	Std. (factor loading)	SMC(R^2^)	CR	AVE
Knowledge	RA1	1.000				0.573	0.328	0.763	0.392
	RA3	1.892	0.099	19.189	***	0.590	0.348		
	RA4	1.259	0.062	20.248	***	0.646	0.417		
	RA6	1.737	0.083	20.879	***	0.687	0.472		
	RA7	1.381	0.069	19.965	***	0.630	0.397		
Severity	RB1	1.000			***	0.748	0.560	0.942	0.767
	RB3	1.092	0.025	43.677	***	0.899	0.808		
	RB4	1.077	0.025	42.558	***	0.879	0.773		
	RB5	1.155	0.026	44.836	***	0.920	0.846		
	RB6	1.089	0.024	44.874	***	0.920	0.846		
Barriers	RC1	1.000			***	0.817	0.667	0.872	0.695
	RC2	1.082	0.026	41.513	***	0.904	0.817		
	RC3	0.970	0.025	38.169	***	0.775	0.601		
Benefit	RD2	1.000			***	0.940	0.884	0.961	0.860
	RD3	1.006	0.011	90.563	***	0.954	0.910		
	RD5	0.972	0.012	80.785	***	0.926	0.857		
	RD1	1.005	0.014	70.472	***	0.889	0.790		
Action	RE1	1.000			***	0.748	0.560	0.953	0.745
	RE4	1.035	0.027	38.210	***	0.796	0.634		
	RE5	1.170	0.028	42.185	***	0.866	0.750		
	RE6	1.173	0.028	42.170	***	0.866	0.750		
	RE7	1.180	0.026	45.936	***	0.931	0.867		
	RE8	1.156	0.027	43.403	***	0.887	0.787		
	RE9	1.160	0.025	45.906	***	0.930	0.865		

**Table 5:** Discriminant validity

	AVE	Action	Benefits	Barriers	Severity	Knowledge
Action	0.745	**0.863**				
Benefits	0.860	0.805	.**927**			
Barriers	0.695	0.030	0.001	**0.834**		
Severity	0.767	0.565	0.616	− 0.086	**0.876**	
Knowledge	0.392	0.207	0.131	0.136	0.154	**0.626**

**Table 6:** Unstandardized parameter estimates

Sub-constructs	Path	Construct	Unstd.	S.E.	C.*R*.	*P*
Knowledge	<---	SRB	1.000			
Severity	<---	SRB	11.813	1.731	6.825	< 0.001
Benefit	<---	SRB	14.038	2.057	6.826	< 0.001
Barriers	<---	SRB	0.100	0.476	0.210	0.833
Action	<---	SRB	13.067	1.881	6.948	< 0.001

**Fig. 1:**
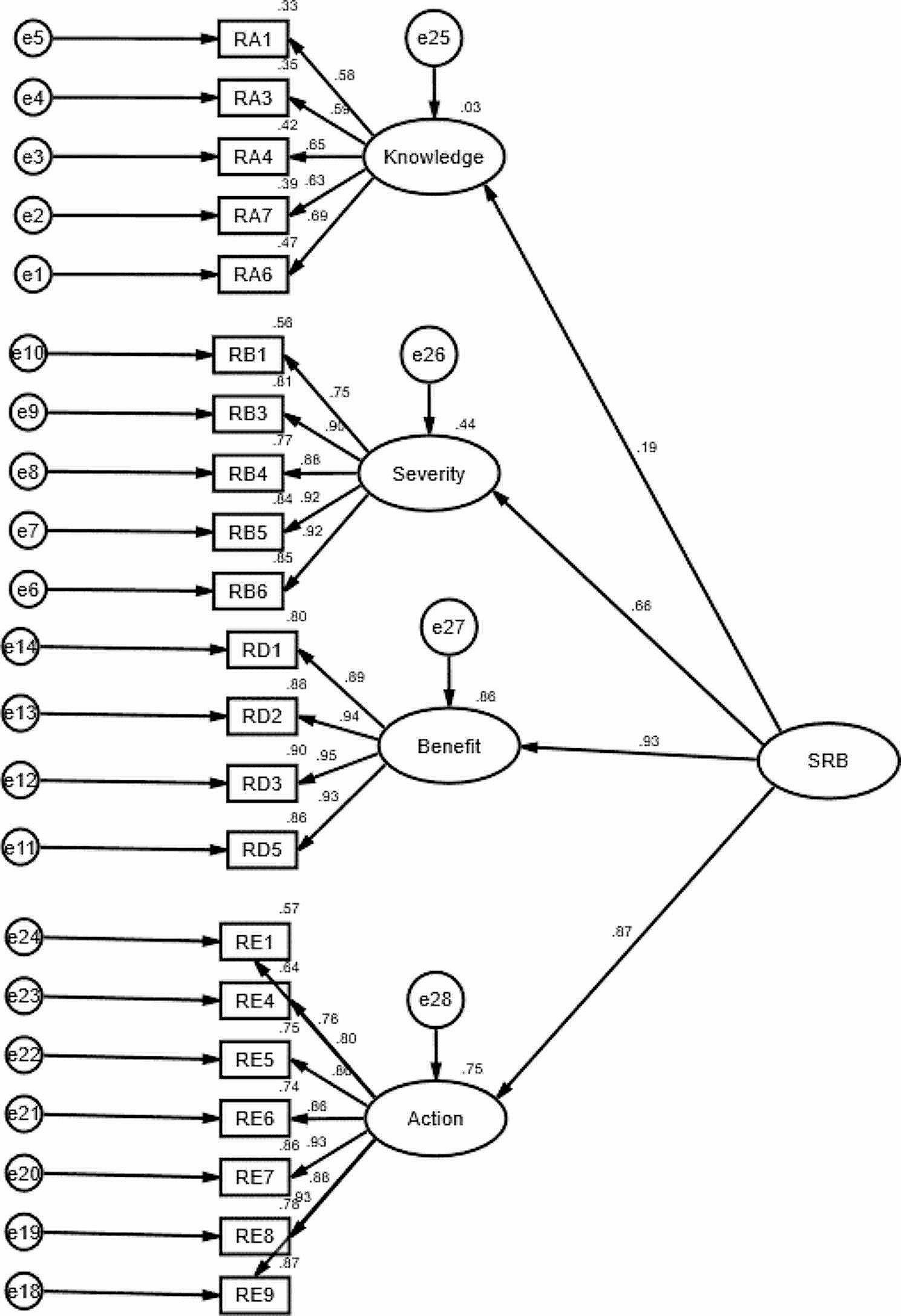
Structural Equation Model (SEM) for Salt-Reduction Behavior (SRB)

### Confirmatory factor analysis

A second-order confirmatory factor analysis (CFA) was evaluated using AMOS 23.0 version, see in Table 6, the construct of barriers was insignificant (*P* = 0.833) which indicates the construct of barriers was not related to SRB. The second-order CFA was conducted to test whether the five constructs belonged to a single broader latent factor of hypertensive patient’s salt-reduction behavior (SRB), after deleting the unrelated construct the second- order CFA results are presented in Fig. 1, the results showed that SRB loads well on its four sub-constructs, the factor loading of SRB on knowledge and severity and benefits and action are 0.186,0.66,0.928 and 0.868 respectively. The R^2^ for all the sub-constructs are 0.03,0.44,0.86, and 0.75, which reflect the contribution of SRB on its four sub-constructs. The final version of the SRB consists of four constructs knowledge, severity, benefits and action.

### Model fit

In this study, relative or incremental fit indexes reflecting the improvement in fit of one model over an alternative (i.e., ratio of chi-square to degrees of freedom, normed fit index (NFI), and target coefficient) are used to compare models. Absolute indexes of goodness-of-fit such as chi-square, goodness-of-fit index (GFI), adjusted goodness-of-fit (AGFI), and root mean square error of approximation (RMSEA), and comparative fit index (CFI) are used to evaluate individual models. For sample size with 75–200 cases, chi-square is a reasonable measure of fit. But for models with more cases, the chi-square is almost always statistically significant. Thus, the chi-square statistic must be interpreted with caution in most applications. Many researches interpret GFI and AGFI scores in the 0.80 to 0.89 range as representing reasonable fit; scores of 0.90 higher are considered evidence of good fit. For RMSEA, a good model has an RMSEA of 0.50 or less. Models whose RMSEA is.10 or more indicate poor fit. The comparative fit index (CFI) analyzes the model fit by examining the discrepancy between the data and the hypothesized model. CFI values range from 0 to 1, with larger values indicating better fit. It is considered very good if it is equal to or greater than 0.95, good between 0.9 and 0.95, suffering between 0.8 and 0.9 and bad if it is less than 0.8 [21]. The ration of chi-square to the degrees of freedom provides information on the relative efficiency of competing models in accounting for the data. Researcher have recommended using ratios as low as 2 or as high as 5 to indicate a reasonable fit [22]. The NFI assesses the fit of a model relative to the fit of a null model by scaling the chi-square value from 0 to 1 with large values indicating better models [23]. The target coefficient index. The goodness-of fit indexes for the first-order model and second-order model and null model are summarized in Table 7. The purpose of the null model is to establish the zero-point for the NFI. As expected, the null model provides a poor fit to the data. Model 1 hypothesizes that all the four sub-constructs are uncorrelated, all the goodness- of- fit indexes indicate the model 1 is not a good fit with sample data.

Model 2 hypothesizes that the four first-order constructs are correlated with each other, Doll and Torkzadeh clearly lay a foundation for this model [24]. Model 2 shows good model-data fit, as indicated by absolute indexes (GFI, AGFI, CFI, RMSEA), and provide substantial improvement over model 1 as evidence by the changes in the NFI index (from 0.9 to 0.919),and the ratio of chi-square to degrees of freedom(from 17.23 to 5.58).

Model 3 hypothesizes that the four first-order constructs belong to one second-order construct. According to Doll and Torkzadeh, if the first-order factors are correlated, it is possible that the correlations between first-order factors is statistically caused by a single second-order factor (Tanaka and Huba 1984). Model 3 shows reasonable model-data fit, as indicated by absolute indexes (GFI, AGFI, CFI, RMSEA). As expected for a second-order model, Model 3’s AGFI CFI and RMSEA scores are slightly lower than its first-order counterpart (Model 2). Like model 2, it provides substantial improvement over model 1, as evidenced by the change in the NFI (from 0.9 to 0.967) and the ratio of chi-square to degrees of freedom (from 17.23 to 5.826). Thus, the results suggest that both Model 2 and Model 3 are satisfactory and competing representation of the underlying structure of the instrument.

The target coefficient was used to test for the existence of a higher-order salt- restriction- behavior (SRB) construct. Using model 2 as the target model, the target coefficient is the ratio of the chi-square of a Model 2 to the Model 3. In this case, a target coefficient of .95 provides reasonable evidence of a second- order SRB construct. 95% of the variation in the four first-order factors in Model 2 is explained by Model 3’ SRB construct.

**Table 7:** Goodness- fit – index for alternative models(*n* = 2082)

Model	Chi-square	Chi-square/df	NFI	GFI	AGFI	CFI	RMSEA
Null model	43542.776	157.764	0	0	0.124	0	0.274(0.272–0.277)
Model 1 (first-order factors uncorrelated)	4341	17.23	0.9	0.859	0.832	0.905	0.088(0.086–0.091)
Model 2 (first-order correlated)	1350	5.58	0.919	0.931	0.931	0.974	0.047(0.044–0.049)
Model 3(second-order factor)	1438	5.826	0.967	0.941	0.928	0.972	0.048(0.046–0.051)

## Discussion

The salt-restriction-behavior scale (SRBS) has emerged as a valuable tool for assessing salt-restriction behavior among Chinese hypertensive patients in Beijing. This scale’s development and validation process involved careful consideration of its structure and content. With a final version comprising 21 items organized into four distinct sub-constructs, the SRBS was rigorously tested for reliability and validity before applying Confirmatory Factor Analysis (CFA) to further assess its suitability and confirm its underlying theoretical framework.

CFA, a powerful statistical technique, was employed to examine if the SRBS’s structure aligns with theoretical expectations. As described by Malhotra [25], CFA aims to validate whether the number of factors and their loadings on observed variables correspond to the expected theoretical model. The results of our CFA strongly supported Model 2 and Model 3, both of which demonstrated superior fit to the data when compared to Model 1, which hypothesized five unrelated constructs. This finding carries significant practical implications for representing salt-restriction behavior effectively.

The support for the higher-order model with four sub-constructs underscores the notion that SRB can be best explained by these specific components. Notably, our research indicated that the construct of perceived barriers to practicing salt-restriction was not significantly related to SRB. Several plausible explanations for this finding exist, including the possibility that the items designed for this construct may not effectively capture the true nature of perceived barriers or that the participants in our study did not perceive any significant obstacles to practicing salt-restriction.

Analyzing the second-order CFA results, we observed that the construct of perceived benefit exhibited the highest factor loading at 0.93, closely followed by the construct of cue to action at 0.87. In contrast, the construct of perceived severity of hypertension showed moderate factor loading, while the construct related to knowledge about hypertension and salt-intake had the lowest factor loading. This suggests that perceived benefit and cues to action play crucial roles in influencing salt-restriction behavior, whereas knowledge about hypertension and salt-intake is less influential.

The lower importance of knowledge as a factor in SRB may be attributed to various factors, such as most participants already possessing knowledge about hypertension and salt-intake due to widespread health education initiatives in China since 2007 [26]. Additionally, the demographic composition of our study, which largely consisted of retired individuals, may have naturally predisposed them to focus on healthy lifestyle knowledge. Since the cohort has a substantial number of retired individuals, the understanding and assessing salt-restriction behavior among hypertensive Chinese patients would be limited to such individuals in this study. However, considering that the typical onset of hypertension occurs predominantly in older adults, our sample composition is reflective of the general hypertensive population in China. The retirement age in China is 55 for women and 60 for men, which aligns with the age group most affected by hypertension. Thus, our findings remain relevant and applicable to the broader hypertensive population.

In conclusion, the SRBS, validated through CFA, offers a valuable framework for understanding and assessing salt-restriction behavior among hypertensive Chinese patients. The identification of specific sub-constructs, as well as the differential impact of knowledge, perceived benefit, and cues to action, sheds light on the intricacies of salt-restriction behavior in this population, providing valuable insights for future research and public health interventions.

## Data Availability

The data supporting the findings of this study are available from the corresponding author upon reasonable request. The data are anonymized and comply with data protection regulations.
